# Variation in sleep profiles in children with ADHD and associated clinical characteristics

**DOI:** 10.1111/jcpp.13835

**Published:** 2023-06-04

**Authors:** Emma Sciberras, Harriet Hiscock, Samuele Cortese, Stephen P. Becker, Julian W. Fernando, Melissa Mulraney

**Affiliations:** ^1^ School of Psychology Deakin University Geelong Vic. Australia; ^2^ Murdoch Children's Research Institute Parkville Vic. Australia; ^3^ Department of Paediatrics University of Melbourne Parkville Vic. Australia; ^4^ The Royal Children's Hospital Parkville Vic. Australia; ^5^ Centre for Innovation in Mental Health School of Psychology, Faculty of Environmental and Life Sciences University of Southampton Southampton UK; ^6^ Clinical and Experimental Sciences (CNS and Psychiatry), Faculty of Medicine University of Southampton Southampton UK; ^7^ Solent NHS Trust Southampton UK; ^8^ Hassenfeld Children's Hospital at NYU Langone New York University Child Study Center New York NY USA; ^9^ Division of Psychiatry and Applied Psychology School of Medicine University of Nottingham Nottingham UK; ^10^ Division of Behavioral Medicine and Clinical Psychology Cincinnati Children's Hospital Medical Center Cincinnati OH USA; ^11^ Department of Pediatrics University of Cincinnati College of Medicine Cincinnati OH USA

**Keywords:** Attention‐deficit/hyperactivity disorder, bedtime, comorbidity, sleep

## Abstract

**Background:**

Sleep difficulties are common in children with attention‐deficit/hyperactivity disorder (ADHD). However, sleep problems are multifaceted and little is known about the variation in sleep difficulties across children with ADHD. We examined the profiles of sleep difficulties in children with ADHD and associated clinical factors (e.g. co‐occurring mental health conditions, stimulant use and parent mental health).

**Methods:**

Data from two harmonised studies of children with ADHD (total: *N* = 392, ages 5–13 years) were used. Parents completed measures of children's sleep, co‐occurring mental health conditions and their own mental health. Both parents and teachers completed measures of child ADHD symptoms and emotional and conduct symptoms. Latent profile analysis was used to identify sleep profiles, and multinomial logistic regression assessed clinical correlates of the groups.

**Results:**

Five sleep profiles were identified: (a) insomnia/delayed sleep phase (36%), (b) generalised sleep difficulties at sleep onset and overnight (25%), (c) high anxious/bedtime resistance difficulties (11%), (d) overnight sleep difficulties including obstructive sleep apnoea and parasomnias (5%) and (e) no sleep difficulties (22%). Compared with the group without sleep difficulties, the generalised, anxious/bedtime resistance and insomnia/delayed sleep phase sleep had greater parent‐reported emotional and conduct symptoms, co‐occurring anxiety and increased parent mental health difficulties. The generalised and anxious/bedtime resistance groups also had greater parent‐reported ADHD symptoms, with the anxious/bedtime resistance sleep group also having more frequent co‐occurring depression and teacher‐reported emotional symptoms.

**Conclusions:**

The sleep difficulties experienced by children with ADHD are varied. Supports to help children with ADHD need to consider the particular profiles of sleep difficulties experienced and broader clinical characteristics. Tailored intervention approaches are likely needed (including a need to address parent mental health).

## Introduction

Sleep difficulties affect up to 70% of children with attention‐deficit/hyperactivity disorder (ADHD; Sung, Hiscock, Sciberras, & Efron, 2008) and are associated with co‐occurring mental health difficulties and increased academic challenges (Langberg et al., 2020; Sung et al., 2008), as well as poorer quality of life (Craig, Weiss, Hudec, & Gibbins, 2020; Sung et al., 2008). Most studies examining sleep in children with ADHD focus on global or average (group level) sleep difficulties (Becker, 2020). However, sleep and hence sleep difficulties are multifaceted and heterogeneous and little is known about the *profiles* of sleep difficulties in children with ADHD, and whether children with ADHD experience difficulties across multiple areas of sleep. A better understanding of the profiles or patterns of sleep difficulties in children with ADHD and associated clinical characteristics may inform further more personalised approaches to support children with ADHD and their families.

A small number of studies have examined the presence of different types of sleep difficulties in children with ADHD. One study of 262 young adolescents with ADHD used a threshold of ≥2, equating to a score of at least ‘sometimes’, to define the presence of a sleep difficulty on each subscale of the parent‐reported Children's Sleep Habits Questionnaire (CSHQ; Langberg et al., 2020). This study found a low prevalence of specific sleep difficulties, with the highest being for daytime sleepiness (27.8%), followed by sleep duration difficulties (5.8%), night waking (5.3%), bedtime resistance (4.1%), sleep anxiety (3.4%), parasomnias (3.0%) and sleep‐disordered breathing (1.5%). Another recent large‐scale population‐based study reported the prevalence of insomnia and delayed sleep–wake phase syndrome to be 29% and 9%, respectively, in children with ADHD (Hysing et al., 2022). However, neither of these studies examined the likely combination of different types of sleep difficulties in children with ADHD.

Another study identified five sleep phenotypes in 30 children with ADHD including (a) narcolepsy‐like; (b) delayed sleep onset insomnia; (c) obstructive sleep apnoea; (d) periodic limb movements; and (e) sleep epileptiform discharges (Miano et al., 2019). This study involved comprehensive sleep assessments including blood tests, video polysomnography, actigraphy and multiple sleep latency tests (Miano et al., 2019). However, the study had a small sample size, had less focus on behavioural sleep difficulties and did not examine predictors of sleep phenotypes. We are unaware of any larger‐scale studies that have examined the presence and co‐occurrence of different profiles of sleep difficulties and associated clinical characteristics in children with ADHD, with a particular emphasis on sleep difficulties that can be supported using behavioural strategies. Previous research has found that the most common sleep difficulties reported by parents include difficulty falling asleep, bedtime resistance and daytime sleepiness (Sung et al., 2008).

Moreover, studies examining factors associated with sleep difficulties in children with ADHD largely focus on the factors associated with global or average sleep difficulties. Less is known about how clinical factors may be differentially associated with different sleep profiles. Corkum and colleagues used factor analysis to assess the presentation of different sleep difficulties in children with ADHD taking medication, children with ADHD not taking medication, a clinical comparison group and a nonclinical comparison group (Corkum, Moldofsky, Hogg‐Johnson, Humphries, & Tannock, 1999). They found that sleep difficulties could be categorised as dyssomnias, sleep‐related involuntary movements and parasomnias, with oppositional defiant disorder and medication use being the strongest predictor of dyssomnias. ADHD‐combined presentation and separation anxiety disorder were the strongest predictors of involuntary overnight movements (Corkum et al., 1999). Other studies have also found that ADHD severity, co‐occurring conditions and medication use are associated with global sleep difficulties in children with ADHD (Becker, Langberg, & Evans, 2015; Chen, Wardlaw, & Stein, 2019; Lycett, Sciberras, Mensah, & Hiscock, 2014). Furthermore, parent mental health difficulties have also been associated with overall sleep difficulties in children with ADHD (Martin, Papadopoulos, Rinehart, & Sciberras, 2021). These studies provide important insights into the factors associated with global sleep difficulties in children with ADHD but do not examine whether clinical factors are associated with different patterns of sleep problems.

In summary, children with ADHD have an increased risk of sleep difficulties relative to those without ADHD; however, less attention has been placed on the different profiles of sleep difficulties experienced, as well as the factors associated with such profiles. Through a better understanding of sleep profiles and associated clinical characteristics, we can refine supports to better suit the needs of children with ADHD and their families. This is important as although there is growing evidence that sleep supports are helpful in children with ADHD, between 28% and 36% of children with ADHD do not respond to a brief behavioural sleep intervention (Hiscock et al., 2019), suggesting that adjustments to interventions based on profiles of sleep difficulties and clinical correlates could further increase the effectiveness of supports. Therefore, this study aimed to examine: (a) the patterns or profiles of sleep difficulties in children with ADHD; and (b) the clinical factors (e.g. co‐occurring conditions and medication use) associated with sleep profiles.

## Methods

### Study design

This study used baseline data from the Attention to Sleep cohort (Lycett, Sciberras, Mensah, Gulenc, & Hiscock, 2014), which included children with ADHD and (a) moderate–severe sleep difficulties (see Measures below) who were enrolled in a randomised controlled trial (RCT) to treat sleep difficulties using a behavioural intervention; and (b) no/mild sleep difficulties. The recruitment of participants into this study has been previously described (Lycett, Sciberras, Mensah, Gulenc, et al., 2014; Lycett, Sciberras, Mensah, & Hiscock, 2014) and is summarised below.

### Ethical considerations

This study was approved by The Royal Children's Hospital Human Ethics (#31193; 30033) and the Department of Education and Early Childhood Development (2011_001307; 2010_000573) ethics committees.

### Participants and recruitment

Participants from the two studies forming the Attention to Sleep cohort were recruited via the same 21 paediatric practices in Victoria, Australia. All methods were the same across the two studies, as described below. Potential participants were mailed information about the study and eligibility was established over the telephone with the primary caregiver (referred to as *parent* throughout). To be sent information about the study, children needed to have been seen at the practice in the last 12 months, be between the age of 5–12 years and to have a diagnosis of ADHD (Lycett, Sciberras, Mensah, Gulenc, et al., 2014; Lycett, Sciberras, Mensah, & Hiscock, 2014).

Children and their families were eligible to participate if at the time of the telephone call they were aged between 5 and 13 years. Children needed to have at least six inattention and/or six hyperactivity/impulsivity symptoms occurring often/very often, assessed using the ADHD Rating Scale – IV (DuPaul, Power, Anastopoulos, & Reid, 1998). In addition, children needed to have been diagnosed with ADHD by their paediatrician. Study‐designed questions were then used to assess whether symptoms were present for at least 6 months, occurred before the age of 7 and occurred across settings.

Children were ineligible if they had suspected obstructive sleep apnoea (OSA) assessed using the OSA subscale from the CSHQ, or if their parent was non‐English speaking, as all study materials were in English. Additionally, children were ineligible if they had a serious medical condition (e.g. severe cerebral palsy), an intellectual disability or if they were receiving specialised sleep assistance from a professional aside from their paediatrician.

Participants (*N* = 392 children, 244 enrolled in the RCT with moderate/severe sleep difficulties, 148 with no/mild sleep difficulties and 70% enrolment rate of those eligible) were recruited between August 2010 and October 2012. Those participating versus those not participating (168 who were eligible but did not return enrolment surveys and consent forms) did not differ in terms of child sex, age or neighbourhood socioeconomic status (Lycett, Sciberras, Mensah, & Hiscock, 2014).

### Sleep measures

#### Parent‐reported sleep difficulties

At the eligibility screen, parents were asked ‘Has your child's sleep been a problem for you over the past 4 weeks?’ and, if yes, were asked to rate whether the sleep problem was mild, moderate or severe (Sung et al., 2008). Moderate–severe sleep difficulties as rated on this question have been associated with poorer outcomes in the general population (Quach, Hiscock, Canterford, & Wake, 2009) and in children with ADHD (Sung et al., 2008). Using this single‐item question, children with ADHD and moderate/severe sleep difficulties have been found to sleep 30 min less per day, have longer sleep onset latency and have more night awakenings using sleep diaries compared to those with no/mild sleep difficulties (Lycett, Mensah, Hiscock, & Sciberras, 2015). Furthermore, children with ADHD and moderate/severe sleep difficulties had substantially higher sleep difficulties on the CSHQ compared to children with ADHD and no/mild sleep difficulties, with an effect size difference of 1.1, 95% CI 1.2 to 0.9 (Lycett et al., 2015).

#### Children's sleep habits questionnaire

Parents completed the CSHQ, a widely used and validated measure assessing difficulties initiating and maintaining sleep (Owens, Spirito, & McGuinn, 2000). The 33‐item measure comprises eight subscales including Bedtime Resistance, Sleep Onset Delay, Sleep Duration, Sleep Anxiety, Night Wakings, Parasomnias, Sleep‐Disordered Breathing and Daytime Sleepiness. Each item is rated on a 3‐point scale with higher scores indicating greater sleep difficulties. There was adequate to very good internal consistency across the CSHQ subscales in our study, as previously reported (Lycett, Sciberras, Mensah, Gulenc, et al., 2014).

### Clinical measures

See Table 1 for a summary of the clinical measures used in this study including co‐occurring mental health conditions, ADHD severity, emotional and conduct symptoms, medication use, parent mental health and sociodemographic factors.

**Table 1:** Clinical measures

Construct	Measure
Co‐occurring depression, anxiety or externalising condition	Anxiety Disorders Interview Schedule – IV (ADIS‐C; Silverman, Saavedra, & Pina, 2001) – completed via telephone with parents. Children were categorised as having a depressive disorder if they met criteria for dysthymia or major depression. An externalising disorder was endorsed if the child met criteria for either ODD or conduct disorder. To meet criteria for an anxiety disorder, two or more anxiety disorders needed to be endorsed as this threshold provides high specificity and sensitivity for identifying anxiety in children with ADHD (Mennin, Biederman, Mick, & Faraone, 2000)
Co‐occurring autism	Parent‐reported diagnosis (yes/no)
ADHD symptoms	ADHD Rating Scale – IV (DuPaul et al., 1998) – parent‐ and teacher‐reported Inattention (9‐items) and hyperactivity/impulsivity (9‐items) subscales
Emotional and conduct symptoms	Strengths and Difficulties Questionnaire (SDQ; Goodman, 2001) – parent‐ and teacher‐reported emotional (5‐items) and conduct (5‐items) subscales
Medication for ADHD	Parents reported whether their child was taking a medication for ADHD including both stimulant and nonstimulant medications (yes/no)
Parent mental health	Depression Anxiety and Stress Scale (Lovibond & Lovibond, 1995) – total score (21 items)
Sociodemographic factors	Child's age and sex, and family socioeconomic status based on residential postcode (Australian Bureau of Statistics, 2006)

### Statistical analysis

Descriptive statistics were used to describe the characteristics of the sample. To determine different patterns of sleep difficulties in children with ADHD (aim 1), we used latent profile analysis which included all subscales from the CSHQ as separate variables in the model. CSHQ scores were converted to z scores given that each of the subscales has a different range of possible scores. To determine the most appropriate number of profiles, a two‐profile model was initially fitted, with subsequent models adding one profile up to seven profiles. The bootstrap likelihood ratio test (BLRT) was used to determine whether each subsequent model was a significantly better fit than the prior model; however, as this test often indicates improved fit up to a high number of profiles, we used multiple considerations to choose the best fitting model. Lower values on the Bayesian Information Criterion (BIC), Akaike Information Criterion (AIC) and the sample‐size‐adjusted BIC (SABIC) are suggestive of a better fitting model (Sinha, Calfee, & Delucchi, 2021), so we used an elbow plot to visualise these scores and assess when model fit was no longer improving with the addition of more profiles (Morin & Marsh, 2015; Sinha et al., 2021). Entropy was also taken into account, which indicates the accuracy of group allocation (Sinha et al., 2021; Wang, Deng, Bi, Ye, & Yang, 2017). We also considered the clinical applicability of the identified number of profiles (Sinha et al., 2021).

Multinomial logistic regression was used to determine the association between clinical factors and each of the sleep difficulty profiles with the no sleep difficulty group as the reference (aim 2). All continuous clinical variables were standardised to aid interpretation. Unadjusted analyses were undertaken followed by adjusted analyses accounting for child age, sex and family socioeconomic status. Analyses were conducted in MPlus (version 8.4, Muthén & Muthén, Los Angeles, CA) Version 8.4 and Stata (version 16.0, StataCorp LLC, College Station, TX).

## Results

### Sociodemographic characteristics

Children were mostly male (85.5%), and 85.2% were taking medication for ADHD (Table 2). On average, children were 10 years of age, with a relatively high rate of co‐occurring conditions.

**Table 2:** Sociodemographic characteristics (*N* = 392)

Characteristic	*N* (%)
Male sex, (yes/no)	335 (85.5)
Child age, *M* (*SD*) range	10.2 (1.9), 5.3–13.5
Medication for ADHD (yes/no)^a^	333 (85.2)
Co‐occurring depression (yes/no)^b^	64 (17.4)
Co‐occurring anxiety (yes/no)^c^	187 (50.5)
Co‐occurring externalising condition (yes/no)^b^	224 (60.9)
Co‐occurring autism (yes/no)	93 (23.7)
Neighbourhood socioeconomic status, *M* (*SD*) range^d^ ^,^ ^e^	1,001.9 (67.3), 816.7–1,117.4

^a^ *n* = 391.

^b^ *n* = 368.

^c^ *n* = 370.

^d^ *n* = 389.

^e^ *M* = 1,000, *SD* = 100.

### Sleep difficulty profiles

All 392 participants had CSHQ sleep data available and were included in the latent profile analyses. We selected a 5‐profile solution as the best fit to the data (Table S1). Although the BLRT indicated that each iteration of the model significantly improved on the previous, there was minimal improvement in model fit after the 5‐profile model. The best log‐likelihood value was not replicated in the 6‐ and 7‐profile models, and these models also included a group comprising <5% of the sample. Although inspection of the elbow plot (Figure S1) showed an elbow at 4‐profiles, we selected 5 profiles based on clinical considerations and excellent accuracy of group allocation (entropy = 0.85). The 5‐profile model included an ‘Overnight Sleep’ difficulties group (described below), which has clinical relevance but was not identified in the 4‐profile model.

**Figure 1:**
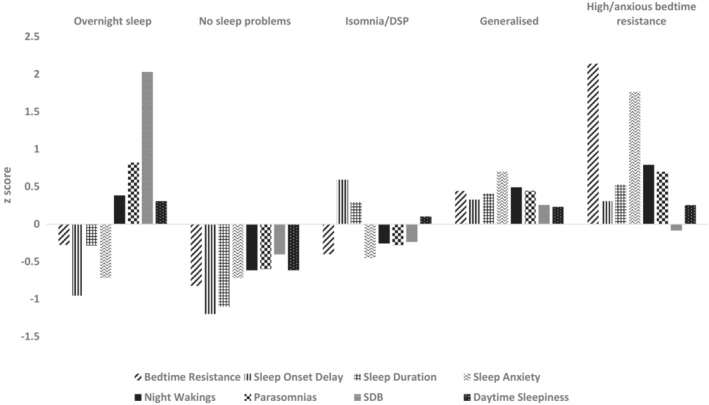
Sleep profiles in children with ADHD. DSP, delayed sleep phase; SDB, sleep‐disordered breathing

Five sleep profiles were identified (Figure 1). The most common was characterised by ‘Insomnia/Delayed Sleep Phase’ difficulties (*n* = 141, 36.0%). Children in this group had Delayed Sleep Onset, Decreased Sleep Duration and slightly elevated Daytime Sleepiness. The next most common group had ‘Generalised’ sleep difficulties (*n* = 98, 25.0%), with sleep difficulties elevated across all subscales, followed by the ‘No Sleep Difficulties’ (*n* = 88, 22.5%) group, with below average sleep difficulties. Eleven per cent had ‘High Anxious/Bedtime Resistance’ difficulties (*n* = 44, 11.2%) with particularly high bedtime resistance and sleep‐related anxiety, and small‐to‐medium elevations across the other domains except for Sleep‐Disordered Breathing. The smallest group comprised those with ‘Overnight Sleep Difficulties’ (*n* = 21, 5.4%) characterised by high Night Wakings, Parasomnias, Sleep‐Disordered Breathing and Daytime Sleepiness, and lower sleep onset difficulties.

### Factors associated with sleep profiles

Given the similarity between unadjusted and adjusted analyses, we report adjusted findings below (see Table 3, see Table S2 for unadjusted results). The sample size for adjusted models ranged from 372 to 389 for parent‐reported variables, 296–297 for teacher‐reported variables and 364–366 for diagnostic interview variables. Table S3 provides descriptive data of the clinical factors by sleep group.

**Table 3:** Clinical factors associated with sleep profiles: Adjusted^a^ analyses

Clinical factors	*N*	No sleep difficulties (ref)	Overnight		Insomnia/DSP		Generalised		High anxious/bedtime resistance	
			RRR (95% CI)	*p*	RRR (95% CI)	*p*	RRR (95% CI)	*p*	RRR (95% CI)	*p*
Inattentive symptoms – P	387	–	1.64 (1.00, 2.71)	.051	1.31 (0.99, 1.72)	.058	1.73 (1.27, 2.35)	<.001	1.67 (1.13, 2.45)	.010
Hyperactivity symptoms – P	389	–	1.68 (1.01, 2.77)	.044	1.26 (0.96, 1.66)	.0990	1.55 (1.15, 2.11)	.005	1.33 (0.90, 1.96)	.149
Inattentive symptoms – T	297	–	0.98 (0.57, 1.69)	.954	0.93 (0.67, 1.28)	.641	0.90 (0.64, 1.26)	.525	1.06 (0.70,1.60)	.797
Hyperactivity symptoms – T	297	–	0.87 (0.50, 1.50)	.618	1.07 (0.78, 1.47)	.666	0.98 (0.69, 1.37)	.887	1.04 (0.6, 1.57)	.861
Emotional symptoms – P	389	–	2.65 (1.56, 4.50)	<.001	1.62 (1.18, 2.22)	.003	3.38 (2.35, 4.86)	<.001	3.22 (2.07, 4.99)	<.001
Conduct symptoms – P	389	–	1.71 (1.05, 2.78)	.031	1.41 (1.06, 1.88)	.020	1.39 (1.02, 1.90)	.037	1.62 (1.10, 2.39)	.015
Emotional symptoms – T	296	–	1.62 (0.97, 2.70)	.065	1.05 (0.75, 1.46)	.779	1.12 (0.79, 1.60)	.523	1.62 (1.07, 2.44)	.021
Conduct symptoms – T	296	–	0.95 (0.55, 1.62)	.844	1.05 (0.77, 1.44)	.745	0.87 (0.62, 1.23)	.440	0.78 (0.51, 1.20)	.262
Medication for ADHD	387	–	0.42 (0.13, 1.42)	.162	0.82 (0.36, 1.87)	.636	0.74 (0.31, 1.76)	.493	0.52 (0.19, 1.43)	.208
Co‐occurring depression	364	–	1.87 (0.51, 6.91)	.347	1.44 (0.62, 3.36)	.401	2.51 (1.04, 6.01)	.040	3.79 (1.37, 10.51)	.010
Co‐occurring anxiety	366	–	3.25 (1.19, 8.83)	.021	1.84 (1.02, 3.33)	.043	6.48 (3.29, 12.75)	<.001	5.85 (2.53, 13.48)	<.001
Co‐occurring externalising	364	–	0.88 (0.33, 2.33)	.790	1.60 (0.91, 2.82)	.106	1.97 (1.05, 3.70)	.035	2.96 (1.26, 6.96)	.013
Co‐occurring autism	388	–	1.63 (0.57, 4.66)	.365	0.89 (0.47, 1.70)	.733	1.09 (0.55, 2.18)	.797	0.82 (0.33, 2.05)	.677
Parent mental health	372	–	2.23 (1.32, 3.78)	.003	1.79 (1.24, 2.59)	.002	2.21 (1.50, 3.26)	<.001	3.17 (2.05, 4.90)	<.001

CI, confidence interval; DSP, delayed sleep phase; RRR, relative risk ratio.

^a^ Adjusted for child sex, age and neighbourhood socioeconomic status.

A one standard deviation increase in parent‐reported inattentive symptoms was associated with an increase in the relative risk of being in the Generalised [Relative Risk Ratio (RRR): 1.73, 95% CI 1.27, 2.35] and High Anxious/Bedtime Resistance (RRR: 1.67, 95% CI 1.13, 2.45) groups compared with the No Sleep Difficulties group. A one standard deviation increase in parent‐reported hyperactive/impulsive symptoms was associated with a higher relative risk of being in the Overnight (RRR: 1.68, 95% CI 1.01, 2.77) and Generalised (RRR: 1.55, 95% CI 1.15, 2.11) groups relative to the No Sleep Difficulties group. Teacher‐reported inattention and hyperactivity/impulsive symptoms did not differ between the sleep difficulties groups and the no sleep difficulties group.

A one standard deviation increase in parent‐reported emotional symptoms and parent‐reported conduct symptoms was associated with an increase in relative risk of being in each of the sleep difficulty groups relative to the No Sleep Difficulties group. Teacher‐reported emotional and conduct symptoms did not differ between the sleep difficulty groups and the No Sleep Difficulties group, with the exception that a one standard deviation increase in teacher‐reported emotional symptoms was associated with increased relative risk of being in the High Anxious/Bedtime Resistance (RRR: 1.62, 95% CI 1.07, 2.44, *p* = .021) group relative to the No Sleep Difficulties group.

ADHD medication use and co‐occurring autism did not significantly vary between the sleep difficulties groups and the No Sleep Difficulties group. Children with co‐occurring depression had a higher relative risk of being in the Generalised (RRR: 2.51, 95% CI 1.04, 6.01) and High Anxious/Bedtime groups (RRR: 3.79, 95% 1.37, 10.51, *p* = .010) compared with the No Sleep Difficulties group. Children with co‐occurring anxiety had a higher relative risk of being in all sleep difficulties groups compared with the No Sleep Difficulties group (*p* ≤ .043). Children with a co‐occurring externalising condition had a higher relative risk of being in the Generalised (RRR: 1.97, 95% CI 1.05, 3.70) and High Anxious/Bedtime Resistance (RRR: 2.96, 95% CI 1.26, 6.96) groups relative to the No Sleep Difficulties group. A one standard deviation increase in parent mental health symptoms was associated with increased relative risk of being in each of the sleep groups compared with the No Sleep Difficulties group.

## Discussion

We explored for the variability of a broad range of sleep difficulties experienced by children with ADHD and the clinical factors associated with different sleep profiles. We identified five sleep profiles in children with ADHD with the largest having Insomnia/Delayed Sleep Phase difficulties (36%), followed by Generalised Sleep difficulties (25%). The identified sleep profiles demonstrated that for many children a number of different sleep difficulties tend to cluster together, for example, anxiety and bedtime resistance. Several clinical factors were associated with the sleep profiles including parent‐reported inattention and hyperactivity symptoms, parent‐reported emotional and conduct symptoms, teacher‐reported emotional symptoms, co‐occurring externalising and internalising conditions, and parent mental health difficulties. Teacher‐reported emotional and conduct symptoms were largely not associated with sleep profiles.

Together, the Generalised and High Anxious/Bedtime Resistance sleep groups comprised 36% of the sample and had the largest number of associated clinical characteristics. Both groups were differentiated from the No Sleep Difficulties group by parent‐reported ADHD symptom severity, parent‐reported emotional and conduct symptoms, co‐occurring anxiety and externalising conditions, and increased parent mental health difficulties. In addition, the High Anxious/Bedtime Resistance group was further differentiated by co‐occurring depression and higher teacher‐reported emotional symptoms. The clinical correlates for these groups are largely consistent with studies examining the factors associated with global measures of sleep difficulties in children with ADHD (Becker et al., 2015; Craig et al., 2020; Lycett, Sciberras, Mensah, & Hiscock, 2014). However, we extend previous research by considering both the variability of sleep difficulties and their associated clinical characteristics, which can help in determining the development of new supports and models of support. For example, given the varying degrees of sleep difficulties and associated clinical characteristics identified, approaches to supporting both sleep and other mental health difficulties in children with ADHD may be particularly fruitful.

Approximately one third of children with ADHD had Insomnia/Delayed Sleep Phase difficulties, which is consistent with the recent prevalence estimates (Hysing et al., 2022). Given the lack of elevations in other more behavioural aspects of sleep such as limit setting and anxiety at night time for this group, it is plausible that circadian dysfunctions may be underlying the sleep difficulties in these children (Korman et al., 2020). However, this is speculative and further research incorporating biological measures (e.g. dim light melatonin onset) is needed to verify this. The clinical characteristics differentiating those with Insomnia/Delayed Sleep Phase and No Sleep Difficulties were parent‐reported emotional and conduct symptoms, co‐occurring anxiety and parent mental health difficulties. These findings are consistent with other studies finding associations between anxiety/emotional symptoms and insomnia in adults with ADHD (Fadeuilhe et al., 2021) and global sleep difficulties in children with ADHD (Becker et al., 2015; Lycett, Sciberras, Mensah, & Hiscock, 2014; Martin et al., 2021).

In contrast, a profile characterised by predominantly Overnight Sleep Difficulties was less common, with only 5% (*n* = 21) of the sample affected but again, findings are consistent with the estimates by Langberg and colleagues in young adolescents with ADHD (Langberg et al., 2020). Furthermore, our study excluded children with suspected OSA (*n* = 24). The Overnight Sleep Difficulties group had particularly elevated levels of sleep‐disordered breathing symptoms and parasomnias. The children in this group may benefit from overnight sleep monitoring via polysomnography to monitor sleep architecture and breathing overnight. Parent‐reported hyperactivity symptoms, emotional and conduct symptoms, co‐occurring anxiety and parent mental health difficulties were all elevated in the Overnight Sleep Difficulties group compared with the No Sleep Difficulties group, which is consistent with research demonstrating the daytime consequences of obstructive sleep apnoea (for a review see Nixon, 2019).

The results from this study underscore the importance of considering the profiles of sleep difficulties experienced by children with ADHD rather than only considering sleep difficulties globally. The results have implications for both diagnostic assessments, as well as supporting sleep in children with ADHD. In terms of diagnostic assessment, clinicians conducting an initial assessment of children referred for ADHD should ask about a broad range of sleep aspects, including anxiety and bedtime resistance issues, and daytime sleepiness, not just difficulties falling asleep/insomnia. The variability in sleep difficulties suggests that tailoring will likely be required to support children with ADHD with their sleep. In our trials of behavioural sleep interventions for children with ADHD, the intervention trialled involved the rapid assessment of different types of sleep difficulties and offering supports tailored to the identified sleep difficulty (Hiscock et al., 2015, 2019). However, it can be challenging to determine which strategies to implement first and the appropriate timing for the introduction of additional strategies. Integrated protocols for supporting children with sleep and co‐occurring difficulties, and additionally assisting with parents with their own mental health difficulties, are likely to be important but remain understudied. We previously found that one of the only moderators of sleep treatment outcomes for children with ADHD was parent depression (Sciberras et al., 2020), suggesting that more intensive supports may be needed in cases where parents have their own mental health difficulties.

This study has a number of strengths, including the relatively large and diverse sample of children with ADHD, the detailed measure of sleep and the broad range of parent‐ and teacher‐reported clinical factors. However, there are some limitations, including the focus on parent‐reported measures of sleep rather than objective measures. In addition, the recruitment into the original studies was reliant on parent report of sleep problem severity, and parents may have different definitions of what constitutes whether sleep is a problem or not, and to what degree of severity. Furthermore, the composition of the sample, with more enrolled in the RCT with moderate/severe sleep problems (*n* = 244), compared with the cohort with no/mild sleep difficulties (*n* = 148), means that our sample is more skewed to having sleep problems and is likely not representative of the general population of children with ADHD. Most of the sample were male and were taking medication for ADHD. Given the cross‐sectional nature of this study, it is impossible to determine the direction of association between the clinical factors examined and particular sleep profiles, and it is likely that these associations are bidirectional in nature.

In conclusion, the sleep difficulties experienced by children with ADHD are varied. Supports to help children with ADHD need to consider the profiles of sleep difficulties experienced and associated clinical characteristics. Tailored intervention approaches are likely needed and the utility of stepped‐care approaches to supporting families with sleep and other co‐occurring conditions should be further investigated.

## Supporting information


**Table S1.** Sleep profile model fit characteristics.
**Table S2.** Clinical factors associated with sleep profiles: Unadjusted analyses.
**Table S3.** Clinical factors by sleep profile group.
**Figure S1.** Elbow plot showing AIC, BIC and SABIC values by sleep profile model.
