# Supplementary material for: Variation in sleep profiles in children with ADHD and associated clinical characteristics
Source: J Child Psychol Psychiatry. 2023 Jun 4;64(10):1462–9. doi: 10.1111/jcpp.13835 (PMC10952554; doi:10.1111/jcpp.13835)
Supplement: Supplementary file 1 — Table S1. Sleep profile model fit characteristics. Table S2. Clinical factors associated with sleep profiles: Unadjusted analyses. Table S3. Clinical factors by sleep profile group. Figure S1. Elbow plot showing AIC, BIC and SABIC values by sleep profile model. [file JCPP-64-1462-s001.docx]

**Supporting information

Table S1. Sleep profile model fit characteristics**

|  | **AIC** | **BIC** | **SABIC** | **BLRT** | **p^a^** | **Entropy** |
| --- | --- | --- | --- | --- | --- | --- |
| 2-profile model | 8446.924 | 8546.205 | 8466.881 | -4435.85 | <.001 | 0.833 |
| 3- profile model | 8195.562 | 8330.585 | 8222.704 | -4198.46 | <.001 | 0.841 |
| 4- profile model | 8077.439 | 8248.204 | 8111.766 | -4063.78 | <.001 | 0.841 |
| 5- profile model | 8000.503 | 8207.008 | 8042.014 | -3995.72 | <.001 | 0.852 |
| 6- profile model^b^ | 7935.597 | 8177.844 | 7984.293 | -3948.25 | <.001 | 0.864 |
| 7-profile model^b^ | 7883.328 | 8161.316 | 7939.209 | -3906.8 | <.001 | 0.862 |

AIC = Akaike Information Criterion; BIC = Bayesian Information Criterion; SAIBIC = Sample-Size Adjusted BIC; BLRT = Bootstrap Likelihood Ratio Test. ^a^ p value indicates whether model fit improved compared to the previous mode using the BLRT test; ^b^ The best loglikelihood value was not replicated in the 6 and 7-profile models.

**Table S2. Clinical factors associated with sleep profiles: Unadjusted analyses**

| **Clinical factors** | **N** | **No sleep difficulties**  **(ref)** | **Overnight sleep** | | **Insomnia/DSP** | | **Generalised** | | **High anxious/bedtime resistance** | |
| --- | --- | --- | --- | --- | --- | --- | --- | --- | --- | --- |
|  |  |  | **RRR**  **(95% CI)** | **p** | **RRR**  **(95% CI)** |  | **RRR**  **(95% CI)** | **p** | **RRR**  **(95% CI)** | **p** |
| Inattentive symptoms - P | 390 | - | 1.62  (0.99, 2.66) | .056 | 1.28  (0.98, 1.67) | .072 | 1.65  (1.22, 1.22) | .001 | 1.55  (1.07, 2.25) | .021 |
| Hyperactivity symptoms - P | 392 | - | 1.69  (1.03, 2.78) | .039 | 1.26  (0.96, 1.65) | .091 | 1.63  (1.21, 2.19) | .001 | 1.49  (1.03, 2.17) | .034 |
| Inattentive symptoms – T | 299 | - | 0.96  (0.57, 1.63) | .887 | 0.92  (0.67, 1.25) | .585 | 0.89  (0.64, 1.25) | .514 | 1.06  (0.70, 1.60) | .774 |
| Hyperactivity symptoms - T | 299 | - | 0.84  (0.49, 1.43) | .514 | 1.04  (0.76, 1.42) | .818 | 0.98  (0.70, 1.37) | .889 | 1.06  (0.71, 1.59) | .777 |
| Emotional symptoms - P | 392 | - | 2.65  (1.57, 4.47) | <.001 | 1.61  (1.18, 2.19) | .003 | 3.20  (2.25, 4.53) | <.001 | 2.80  (1.85, 4.23) | <.001 |
| Conduct symptoms - P | 392 | - | 1.79  (1.10, 2.90) | .019 | 1.47  (1.11, 1.95) | .008 | 1.49  (1.10, 2.02) | .010 | 1.78  (1.23, 2.60) | .002 |
| Emotional symptoms - T | 298 | - | 1.64  (0.99, 2.71) | .057 | 1.06  (0.76, 1.47) | .720 | 1.13  (0.80, 1.60) | .490 | 1.61  (1.07, 2.40) | .021 |
| Conduct symptoms - T | 298 | - | 0.98  (0.58, 1.65) | .926 | 1.07  (0.79, 1.46) | .662 | 0.90  (0.64, 1.26) | .527 | 0.81  (0.53, 1.23) | .322 |
| ADHD medication use | 391 | - | 0.41  (0.12, 1.36) | .146 | 0.82  (0.36, 1.84) | .627 | 0.71  (0.30, 1.67) | .433 | 0.50  (0.19, 1.33) | .166 |
| Comorbid depression | 368 | - | 1.96  (0.54, 7.12) | .306 | 1.58  (0.68, 3.63) | .285 | 2.26  (0.96, 5.33) | .062 | 2.96  (1.12, 7.84) | .029 |
| Comorbid anxiety | 370 | - | 3.15  (1.18, 8.41) | .022 | 1.85  (1.04, 3.31) | .038 | 5.59  (2.92, 10.70) | <.001 | 4.72  (2.13, 10.44) | <.001 |
| Comorbid externalising disorder | 364 | - | 0.95  (0.37, 2.48) | .922 | 1.72  (0.99, 2.99) | .055 | 2.06  (1.12, 3.81) | .021 | 3.36  (1.46, 7.69) | .004 |
| Comorbid ASD | 392 |  | 1.50  (0.54, 4.19) | .439 | 0.85  (0.45, 1.58) | .599 | 0.97  (-0.50, 1.90) | .936 | 0.77  (0.32, 1.85) | .562 |
| Parent mental health | 374 |  | 2.20  (1.31, 3.69) | .003 | 1.73  (1.21, 2.49) | .003 | 2.13  (1.46, 3.11) | <.001 | 3.01  (1.98, 4.58) | <.001 |

CI = Confidence Interval; DSP = Delayed Sleep Phase; RRR = Relative Risk Ratio.

**Table S3. Clinical factors by sleep profile group**

| **Clinical factors** | **No sleep difficulties**  **(n=88)^a^** | | **Overnight sleep**  **(n=21)^b^** | | **Insomnia/DSP**  **(n=141)^c^** | | **Generalised**  **(n=98)^d^** | | **High anxious/bedtime resistance**  **(n=44)^e^** | |
| --- | --- | --- | --- | --- | --- | --- | --- | --- | --- | --- |
|  | **M** | **SD** | **M** | **SD** | **M** | **SD** | **M** | **SD** | **M** | **SD** |
| Inattentive symptoms - P | 17.15 | 5.03 | 19.67 | 4.91 | 140 | 18.46 | 19.74 | 5.63 | 19.44 | 4.74 |
| Hyperactivity symptoms - P | 14.90 | 5.40 | 17.80 | 6.10 | 16.22 | 5.23 | 17.61 | 5.91 | 17.16 | 5.61 |
| Inattentive symptoms – T | 15.45 | 7.03 | 15.18 | 6.76 | 14.83 | 7.35 | 14.65 | 7.33 | 15.88 | 7.29 |
| Hyperactivity symptoms - T | 11.42 | 7.31 | 10.19 | 6.87 | 11.68 | 6.81 | 11.25 | 7.35 | 11.84 | 7.70 |
| Emotional symptoms - P | 3.09 | 2.19 | 5.19 | 2.50 | 4.05 | 2.26 | 5.62 | 2.54 | 5.32 | 2.45 |
| Conduct symptoms - P | 3.96 | 2.39 | 5.32 | 2.23 | 4.84 | 2.43 | 4.88 | 2.43 | 5.32 | 2.58 |
| Emotional symptoms - T | 2.83 | 2.43 | 4.08 | 2.93 | 2.96 | 2.16 | 3.11 | 4.03 | 4.03 | 2.88 |
| Conduct symptoms - T | 3.07 | 3.18 | 3.00 | 2.52 | 3.26 | 2.56 | 2.78 | 2.60 | 2.53 | 2.35 |
| Parent mental health symptoms | 22.97 | 18.01 | 37.51 | 26.53 | 31.82 | 26.06 | 36.71 | 21.85 | 47.56 | 27.58 |
|  | **n** | **%** | **n** | **%** | **n** | **%** | **n** | **%** | **n** | **%** |
| ADHD medication use | 78 | 88.64 | 16 | 76.19 | 121 | 86.43 | 83 | 84.69 | 35 | 79.55 |
| Comorbid depression | 9 | 10.71 | 4 | 19.05 | 21 | 15.91 | 19 | 21.35 | 11 | 26.19 |
| Comorbid anxiety | 25 | 29.76 | 12 | 57.14 | 58 | 43.94 | 64 | 70.33 | 28 | 66.67 |
| Comorbid externalising disorder | 41 | 48.81 | 10 | 47.62 | 82 | 62.12 | 59 | 66.29 | 32 | 76.19 |
| Comorbid ASD | 22 | 25.00 | 7 | 33.33 | 31 | 21.99 | 24 | 24.49 | 9 | 20.45 |

^a^ n=64-88; ^b^ n=18-21; ^c^ n=105-141; ^d^ n=74-98; ^e^ n=37-44. DSP = Delayed Sleep Phase.

**Figure S1. Elbow plot showing AIC, BIC and SABIC values by sleep profile model**

Note: AIC = Akaike Information Criterion; BIC = Bayesian Information Criterion; SAIBIC = Sample-Size Adjusted BIC.
